# Evaluating diagnostic accuracy of an RT-PCR test for the detection of SARS-CoV-2 in saliva

**DOI:** 10.1186/s41512-024-00176-2

**Published:** 2024-07-24

**Authors:** Natasha Samsunder, Aida Sivro, Razia Hassan-Moosa, Lara Lewis, Zahra Kara, Cheryl Baxter, Quarraisha Abdool Karim, Salim Abdool Karim, Ayesha B. M. Kharsany, Kogieleum Naidoo, Sinaye Ngcapu

**Affiliations:** 1 Centre for the AIDS Programme of Research in South Africa (CAPRISA), 719 Umbilo Road, Durban, 4001 South Africa; 2 Department of Medical Microbiology, University of KwaZulu-Natal, Durban, South Africa; 3 JC Wilt Infectious Disease Research Centre, National Microbiology Laboratory, Public Health Agency of Canada, Winnipeg, MB Canada; 4 Department of Medical Microbiology and Infectious Diseases, University of Manitoba, Winnipeg, MB Canada; 5 SAMRC-CAPRISA HIV-TB Pathogenesis and Treatment Research Unit, Doris Duke Medical Research Institute, University of KwaZulu-Natal, Durban, South Africa; 6 Centre for Epidemic Response and Innovation (CERI), Stellenbosch University, Stellenbosch, South Africa; 7 Department of Epidemiology, Columbia University, New York, NY USA

**Keywords:** COVID-19, SARS-CoV-2, Saliva, Nasopharyngeal swab

## Abstract

**Background and objective:**

Saliva has been proposed as a potential more convenient, cost-effective, and easier sample for diagnosing SARS-CoV-2 infections, but there is limited knowledge of the impact of saliva volumes and stages of infection on its sensitivity and specificity.

**Methods:**

In this study, we assessed the performance of SARS-CoV-2 testing in 171 saliva samples from 52 mostly mildly symptomatic patients (aged 18 to 70 years) with a positive reference standard result at screening. The samples were collected at different volumes (50, 100, 300, and 500 µl of saliva) and at different stages of the disease (at enrollment, day 7, 14, and 28 post SARS-CoV-2 diagnosis). Imperfect nasopharyngeal (NP) swab nucleic acid amplification testing was used as a reference. We used a logistic regression with generalized estimating equations to estimate sensitivity, specificity, PPV, and NPV, accounting for the correlation between repeated observations.

**Results:**

The sensitivity and specificity values were consistent across saliva volumes. The sensitivity of saliva samples ranged from 70.2% (95% CI, 49.3–85.0%) for 100 μl to 81.0% (95% CI, 51.9–94.4%) for 300 μl of saliva collected. The specificity values ranged between 75.8% (95% CI, 55.0–88.9%) for 50 μl and 78.8% (95% CI, 63.2–88.9%) for 100 μl saliva compared to NP swab samples. The overall percentage of positive results in NP swabs and saliva specimens remained comparable throughout the study visits. We observed no significant difference in cycle number values between saliva and NP swab specimens, irrespective of saliva volume tested.

**Conclusions:**

The saliva collection offers a promising approach for population-based testing.

**Supplementary Information:**

The online version contains supplementary material available at 10.1186/s41512-024-00176-2.

## Introduction

The rapid and accurate identification of severe acute respiratory syndrome coronavirus 2 (SARS-CoV-2) cases is an important strategy for controlling the spread of SARS-CoV-2 viral infection. To date, testing of nasopharyngeal (NP) swab samples for SARS-CoV-2 using the reverse transcriptase-polymerase chain reaction (RT-PCR) remains the reference standard for SARS-CoV-2 detection and diagnosis [1, 2]. However, the collection of NP swab samples suffers from supply chain constraints and is inclined to cause nasal discomfort, with the risk of suboptimal self-sampling, and increased risk of infection transmission via droplets or aerosol particles due to the irritation of the nasal passage [3]. Given these limitations, there is a need for alternative samples, including collection procedures that are less invasive and acceptable to patients and simultaneously produce accurate results.

Saliva samples are an attractive alternative for diagnosis due to ease of collection and patient preference and acceptability. Unlike NP swabs, saliva is most likely to increase compliance from the population for testing and decrease exposure risk to healthcare workers during the collection process [4–6]. Studies have demonstrated that SARS-CoV-2 can be detected from the saliva of coronavirus disease 2019 (COVID-19) patients with sensitivity range from 45 to 97%, with improvement observed after RNA purification in crude sample [7–10]. Data regarding sensitivity for SARS-CoV-2 detection in saliva and NP swab specimens are mixed, ranging from similar [4, 11, 12], increased [13], or decreased [14] sensitivity in saliva compared to NP swabs from COVID-19 patients.

Monitoring SARS-CoV-2 using an accurate, non-invasive, easily accessible collection method remains a public health need. Here, we assessed SARS-CoV-2 detection in paired saliva and NP swab samples collected from both asymptomatic and symptomatic COVID-19 patients attending healthcare facilities in Durban, South Africa. Additionally, we determined the effect of different saliva volumes on the sensitivity of SARS-CoV-2 PCR tests.

## Methods

### Study design, participants, and sample collection

This ancillary study nested in a prospective, observational multicentric study was conducted in the King Dinuzulu Hospital Complex and KwaMashu community health center in Durban, KwaZulu-Natal. Outpatients seeking testing for SARS-CoV-2 infection, irrespective of symptoms, were screened for SARS-CoV-2 using real-time PCR (RT-PCR). Adult patients 18 years old or over that tested positive for SARS-CoV-2 by NP swabs at screening were invited to enroll into the main research study within 3 days of screening (baseline). Following informed consent, enrolled patients completed a questionnaire on basic demographics and clinical data. They were asked to return on day 7, day 14, and day 28 from positive RT-PCR test and provided NP swabs, saliva, demographic, and clinical information. NP swab was collected before saliva samples at the same visits by the study nurse. NP swab was placed into 2 ml viral transport media (VTM) (RPMI-1640 media, 10% fetal bovine serum plus penicillin–streptomycin). Saliva samples were collected by instructing a patient to spit into a sterile container with the obtained volume ranging between 50 and 2000 μl. Depending on the volume of the sample provided, up to 4 different volumes of saliva (50, 100, 300, and 500 μl) were mixed with VTM (topped up to 1100 μl) for SARS-CoV-2 testing. Saliva sensitivity was calculated using the NP RT-PCR as the imperfect reference standard. In addition, salivary specificity was calculated among those who tested positive for SARS-CoV-2 at screening. Study sample size was based on sample and resource availability at the time of the testing and sample collection. All SARS-CoV-2 positive cases were reported to the South African National Department of Health using the National Medical Conditions (NMC) surveillance system. The study formed part of studies approved by the KwaZulu-Natal Biomedical Research Ethics Committee (BREC approval Reference Numbers: BREC/00001195/2020; BREC/00003106/2021 and BREC/00003902/2022). The study follows the Standards for Reporting Diagnostic accuracy studies (STARD) guidelines [15].

### SARS-CoV-2 RT-PCR

Viral RNA was extracted from NP swabs and different volumes of saliva using the Abbott mSample Preparation System (Abbott GmbH & Co, Germany) and RT-PCR for SARS-CoV-2 RNA-dependent RNA polymerase (RdRp) and N genes were run on the Abbott m2000 RealTime System. The instrument automatically reports the results and interpretation on the Abbott m2000*rt* workstation. The Abbott RealTime SARS-CoV-2 positive results are reported with cycle number (CN) values [16]. A cycle number (CN) of < 31 was regarded as a positive result.

### Statistical analysis

Statistical analyses were conducted in GraphPad Prism 8.0.0 (GraphPad Software, San Diego, CA, USA) and SAS version 9.4 (SAS Institute Inc., Cary, NC, USA). Abbott RealTime SARS-CoV-2 assay was used to calculate test performance characteristics (sensitivity, specificity, PPV and NPV). Sensitivity, specificity, PPV, and NPV were estimated using logistic regression with generalized estimating equations (GEE) to account for the correlation between repeated observations, and 95% confidence intervals were estimated using back-transformation [17]. Paired *t*-test was used to compare the CN values between NP swabs and saliva samples.

## Results

### Study participant characteristics

Matching saliva and NP swab sample pairs were collected and tested for SARS-CoV-2 from 52 SARS-CoV-2 participants screened and enrolled in the study over 1–4 time points. Screening and enrolment characteristics for the participants is detailed in Table 1. The majority of participants were female (57.7%, 30/52) and with an age range of 19–78 years. Overall, 63.5% had mild symptoms (33/52); 13.5% had moderate symptoms (7/52), and 23.1% were asymptomatic (12/52). Comorbidities reported were hypertension (17.3%; 9/52), diabetes (13.5%, 7/52), asthma (3.8%, 2/52), cardiac symptoms (1.9%, 1/52), and body mass index (BMI) > 30 (48.1%, 25/52).

**Table 1:** Screening and enrolment characteristics

**Participant characteristics**		**Overall (*N* = 52)** ***n* (%)**
Gender	Male	22 (42.3)
	Female	30 (57.7)
Age group	< 30	7 (13.5)
	30–39	13 (25)
	40–49	11 (21.2)
	50–59	9 (17.3)
	60 +	12 (23.1)
Current tuberculosis	No	50 (96.2)
	Yes	2 (3.8)
Previous tuberculosis	No	45 (86.5)
	Yes	7 (13.5)
Hypertension	No	43 (82.7)
	Yes	9 (17.3)
Diabetes	No	45 (86.5)
	Yes	7 (13.5)
Asthma	No	50 (96.2)
	Yes	2 (3.8)
Cardiac	No	51 (98.1)
	Yes	1 (1.9)
BMI ≥ 30	No	27 (51.9)
	Yes	25 (48.1)
Symptoms at screening	No	5 (9.6)
	Yes	47 (90.4)
COVID-19 severity assessment	Asymptomatic	12 (23.1)
	Mild	33 (63.5)
	Moderate	7 (13.5)

### Performance of saliva samples for SARS-CoV-2 testing

The 2 × 2 tables specifying sample numbers per saliva volume are included in the supplemental materials (Sup. Table 1). We assessed the performance of different saliva volumes against NP swabs. Only participants with a positive reference standard result were enrolled into the study, and thus only sensitivity estimates should be considered. Sensitivity was similar across different saliva volumes, ranging from 70.2% (95% CI, 49.3–85.0%) for 100 μl of saliva to 81.0% (95% CI, 51.9–94.4%) for 300 μl of saliva (Table 2). While all study participants tested positive for SARS-CoV-2 at screening, a number of them cleared the infection by the enrolment visit allowing for specificity estimation. Similar specificity was observed across volumes ranging from 78.8% (95% CI, 62.3–89.3%) for 50 μl of saliva to 86.4% (95% CI, 66.7–95.3%) for 500 μl of saliva compared to NP swab samples.

**Table 2:** Comparison of real-time RT-PCR results of paired saliva and NP swab samples with respective sensitivity and specificity

Saliva volume	*N*	Sensitivity	95% confidence interval	Specificity	95% confidence interval
50 μl	41	73.2	49.5–88.4	75.8	55.0–88.9
100 μl	40	70.2	49.3–85.0	78.6	58.3–90.6
300 μl	33	81.0	51.9–94.4	77.9	56.4–90.6
500 μl	57	70.8	49.0–85.9	78.8	63.2–88.9

**Fig. 1:**
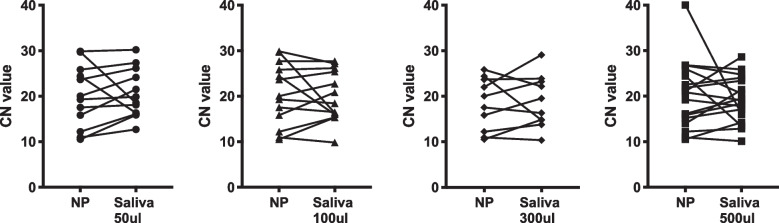
Differences in CN values between paired NP swab and saliva SARS-CoV-2 positive samples across different saliva volumes (50 μl, 100 μl, 300 μl, and 500 μl). Paired *t*-test was used to assess differences in CN values between NP and saliva at specific volumes with no significant differences noted

When we compared CN values of matched SARS-CoV-2 positive saliva and NP swab samples (as an indicator of viral load) across different saliva volumes, we observed no significant difference (Fig. 1). While the percentage of positive NP swabs and saliva specimens was overall similar across different study visits (Sup. Figure 1), saliva showed higher positivity at several time points and volumes. A higher percentage of saliva samples tested positive for SARS-CoV-2 at day 28 post-study enrolment in 300 μl and 500 μl samples compared to NP swab sample; however, the sample size was small at this time point.

## Discussion

The use of an optimal clinical sample is crucial for detecting and monitoring the spread of SARS-CoV-2 within the community. NP swabs have been the traditional choice, but saliva has emerged as a viable and less invasive sample for SARS-CoV-2 detection [4–6]. In this study, we aimed to assess the utility of saliva compared to NP samples for diagnosing SARS-CoV-2 infection at different stages of the disease.

Our results demonstrate moderate to high sensitivity and specificity across different saliva volumes. The sensitivity of saliva testing ranged between 70.2% (100 μl) and 81.0% (300 μl), while the specificity ranged between 75.8% (50 μl) and 78.8% (500 μl) compared to NP swab samples. We observed no major differences in sensitivity and specificity based on different saliva volumes tested. These findings align with previous reports [18–21]. As the detection of SARS-CoV-2 in saliva was comparable to NP swabs, saliva may be a valuable alternative for identifying mild or subclinical infections. The safety of saliva collection by adult patients eliminates the risk of exposure for healthcare workers [22]. Saliva samples would be useful for large scale surveillance programs [23]. While the overall percentage of positive results for both NP swabs and saliva specimens were generally similar up to 28 days post-diagnosis, at several time points and volumes, the detectability of SARS-CoV-2 was slightly higher in saliva samples compared to NP swab. The implications of this on the length of viral shedding and transmissibility are hard to determine due to limited sample size, especially at later time points. A previous study reported that SARS-CoV-2 can often be detected earlier in the saliva compared to nasal passage [24]. However, there is significant variability between individuals as the dynamics of viral shedding at different sites are likely influenced by both viral characteristics as well as host factors and pre-existing immunity of the infected individual [25].

An interesting finding of this study were the comparable CN values between the NP sample and saliva, regardless of the volume of saliva tested. This indicates that using reduced volumes of saliva can still provide valuable information for detecting infection, monitoring its progression, evaluating intervention effectiveness, and assessing viral shedding dynamics. This is important as ability to produce saliva varies between individuals and can be impacted by a number of biological and behavioral factors.

Our study has some limitations that should be considered. Using a single detection system may yield different results compared to other platforms. The viscosity of saliva samples presents challenges for automated dispensing systems. Alternate methods, such as throat washing with normal saline, could potentially improve yield [26, 27]. Additionally, our study’s small sample size and limited geographic scope may introduce several limitations, including limited generalizability of the findings and a lack of precision and reliability. It is also important to note that all study participants tested positive for SARS-CoV-2 at screening, and therefore specificity calculations at the subsequent enrolment visit should be interpreted with caution.

In conclusion, our study adds to a growing body of evidence supporting saliva as a valid and reliable alternative for diagnosing SARS-CoV-2 in patients across all stages of infection. Saliva could be considered a preferred sample in patients, particularly in challenging situations where obtaining proper NP swab sample is difficult. However, a negative saliva result in symptomatic patients may warrant retesting with a different sample type to improve detection rates and reduce false negatives. Further testing, validation, and implementation of saliva-based SARS-CoV-2 diagnostics on different platforms are warranted.

### Supplementary Information

Supplementary Material 1: Supplementary Table 1. 2 × 2 tables for different saliva volumes with NP RT-PCR result as an imperfect reference. Sup. Figure 1. Precent positivity for SARS-CoV-2 in matched NP and saliva samples for total samples and across different study visits: 0 days (enrolment), 7 days, 14 days, and 28 days from positive diagnosis. A) Paired NP and saliva 50 μl samples. B) Paired NP and saliva 100 μl samples C) Paired NP and saliva 300 μl samples and D) Paired NP and saliva 500 μl samples.

## Data Availability

The de-identified patient-level data can be accessed by contacting the corresponding author with a detailed description of the research question.
