# Supplementary material for: Evaluating diagnostic accuracy of an RT-PCR test for the detection of SARS-CoV-2 in saliva
Source: Diagn Progn Res. 2024 Jul 24;8:9. doi: 10.1186/s41512-024-00176-2 (PMC11267770; doi:10.1186/s41512-024-00176-2)
Supplement: Supplementary file 1 — Supplementary Material 1: Supplementary Table 1. 2 × 2 tables for different saliva volumes with NP RT-PCR result as an imperfect reference. Sup. Figure 1. Precent positivity for SARS-CoV-2 in matched NP and saliva samples for total samples and across different study visits: 0 days (enrolment), 7 days, 14 days, and 28 days from positive diagnosis. A) Paired NP and saliva 50 μl samples. B) Paired NP and saliva 100 μl samples C) Paired NP and saliva 300 μl samples and D) Paired NP and saliva 500 μl samples. [file 41512_2024_176_MOESM1_ESM.docx]

**Supplementary Table 1.** 2x2 tables for different saliva volumes with NP RT-PCR result as an imperfect reference

| n=41,  Volume =50μl | | | Nasopharyngeal swab | | |
| --- | --- | --- | --- | --- | --- |
|  | | | Positive | Negative | Total |
| Saliva | Positive | | 12 | 5 | 17 |
|  | Negative | | 4 | 20 | 24 |
|  | Total | | 16 | 25 | 41 |
| n=40,  Volume =100μl | | | Nasopharyngeal swab | | |
|  | | | Positive | Negative | Total |
| Saliva | | Positive | 12 | 4 | 16 |
|  |  | Negative | 5 | 19 | 24 |
|  |  | Total | 17 | 23 | 40 |
| n=33,  Volume =300μl | | | Nasopharyngeal swab | | |
|  | | | Positive | Negative | Total |
| Saliva | Positive | | 10 | 4 | 14 |
|  | Negative | | 2 | 17 | 19 |
|  | Total | | 12 | 21 | 33 |
| n=57,  Volume =500μl | | | Nasopharyngeal swab | | |
|  | | | Positive | Negative | Total |
| Saliva | | Positive | 17 | 7 | 24 |
|  |  | Negative | 7 | 26 | 33 |
|  |  | Total | 24 | 33 | 57 |

**Sup. Figure 1.** Precent positivity for SARS-CoV-2 in matched NP and saliva samples for total samples and across different study visits: 0 days (enrolment), 7 days, 14 days, and 28 days from positive diagnosis. A) Paired NP and saliva 50µl samples. B) Paired NP and saliva 100µl samples C) Paired NP and saliva 300µl samples and D) Paired NP and saliva 500µl samples.
